# Risks of cardiovascular diseases associated with dipeptidyl peptidase-4 inhibitors and other antidiabetic drugs in patients with type 2 diabetes: a nation-wide longitudinal study

**DOI:** 10.1186/s12933-016-0350-4

**Published:** 2016-03-01

**Authors:** Huang-Tz Ou, Kai-Cheng Chang, Chung-Yi Li, Jin-Shang Wu

**Affiliations:** Institute of Clinical Pharmacy and Pharmaceutical Sciences, College of Medicine, National Cheng Kung University, 1 University Road, Tainan, 7010 Taiwan; Department of Public Health, College of Medicine, National Cheng Kung University, Tainan, Taiwan; Department of Public Health, China Medical University, Taichung, Taiwan; Department of Family Medicine, College of Medicine, National Cheng Kung University, Tainan, Taiwan; Department of Family Medicine, National Cheng Kung University Hospital, Tainan, Taiwan

**Keywords:** Type 2 diabetes mellitus, Dipeptidyl peptidase-4 inhibitors, Cardiovascular diseases, Antidiabetic drugs, Hypoglycemia

## Abstract

**Background:**

Several antidiabetic drugs (i.e., sulfonylureas; SU, rosiglitazone) have been reported to be associated with increased risks of cardiovascular diseases (CVD) in patients with type 2 diabetes mellitus (T2DM). Dipeptidyl peptidase-4 inhibitors (DPP4i) are newly available antidiabetic drugs. Most studies only compared DPP4i with a placebo or SU, or targeted a specific CVD event of interest (i.e., heart failure; HF). Comparative research of CVD risks of DPP4i with other antidiabetic drugs (i.e., metformin, thiazolidinediones, meglitinides, acarbose, and insulin) remains scarce. This study was aimed to assess comparative risks of CVD, including ischemic stroke, myocardial infarction (MI) and HF, and hypoglycemia of DPP4i with other antidiabetic drugs.

**Methods:**

We utilized Taiwan’s National Health Insurance Research Database. A total of 123,050 T2DM patients newly prescribed oral antidiabetic treatments were identified in 2009–2010 and followed until 2013. Outcome endpoints included a composite of CVD events: hospitalizations for ischemic stroke, MI and HF, and hypoglycemia. Time-varying Cox proportional hazards regression was applied to assess the time to event hazards of various antidiabetic drugs, adjusted for patients’ demographics, comorbidity, diabetic complications, and co-medications. Additional analyses were performed for the patients with and without CVD history, respectively.

**Results:**

DPP4i users had significantly lower CVD risks as compared to that of non-DPP4i users (adjusted hazard ratio [aHR]: 0.83, 95 % confidence interval [CI]: 0.76–0.91). Compared to DPP4i users, meglitinides (aHR 1.3, 95 % CI 1.20–1.43) and insulin users (aHR 3.73, 95 % CI 3.35, 4.14) had significantly higher risks for composite CVD, as well as those for stroke, MI, HF, and hypoglycemia. Additionally, metformin users had significantly lower risks for composite CVD risk (aHR 0.87, 95 % CI 0.79–0.94), as well as those for MI, HF, and hypoglycemia, as compared to those of DPP4i users. Although there was a trend toward low CVD risks in pioglitazone users, the role of potential confounding by indication cannot be excluded.

**Conclusions:**

DPP4i-treated T2DM patients had lower risks for CVD as compared to those for non-DPP4i users, except metformin users.

**Electronic supplementary material:**

The online version of this article (doi:10.1186/s12933-016-0350-4) contains supplementary material, which is available to authorized users.

## Background

Cardiovascular diseases (CVD) are highly prevalent complications in patients with type 2 diabetes mellitus (T2DM), which are the leading of deaths in such individuals [1]. However, with emerging trials evaluating cardiovascular effects of antidiabetic drugs, not all the drugs appear to reduce CVD risks in T2DM patients. Sulfonylureas (SU) have been reported to be associated with increased CVD risks [2, 3]. The meta-analyses of clinical trials [4] and observational studies [5] showed that rosiglitazone was associated with excess myocardial infarction (MI) and heart failure (HF) risks, although a recent large prospective trial, the RECORD, did not have sufficient data to determine if it yields a higher risk for MI as compared to metformin or SU [6]. Conversely, the cardiovascular benefits have been seen with several antidiabetic drugs: metformin for T2DM patients with overweight [7], acarbose for the patients with impaired glucose tolerance (IGT) [8, 9], and empagliflozin in those at high risk of CVD events [10].

Dipeptidyl peptidase-4 inhibitors (DPP4i) are newly available oral hypoglycemic agents (OHAs). DPP4i suppresses the breakdown of incretin hormones glucagon-like peptide-1 and glucose-dependent insulinotropic peptide, achieving glycemic control. The CVD risks associated with DPP4i treatment have been investigated. DPP4i was shown to not have increased risks for ischemic stroke and MI [11–19]. Recent trials noticed that saxagliptin (SAVOR-TIMI 53 trial [12]) and alogliptin (EXAMINE [13]) had a higher risk for heart failure (HF) as compared to placebo, while a large observational study of 127,555 T2DM patients in Italy showed a significantly lower HF risk of DPP4i as compared with SU [20]. However, most studies only compared DPP4i with a placebo [12, 13] or SU [11, 15, 17, 18, 21], or targeted a specific CVD event of interest (i.e., HF [20]). Research that assesses comparative CVD risks of DPP4i with other antidiabetic drugs (i.e., metformin, thiazolidinediones (TZDs), meglitinides, acarbose, and insulin) remains scarce.

The present study utilizes a comprehensive national cohort of diabetic patients in Taiwan to evaluate the risks of CVD, including stroke, MI and HF, and hypoglycemia associated with DPP4i as compared with those of other antidiabetic drugs.

## Methods

The Institutional Review Board of National Cheng Kung University Hospital approved the study before commencement (A-ER-103-298).

### Data source

We used the Longitudinal Cohort of Diabetes Patients database (LHDB) 1996–2013, retrieved from the National Health Insurance Research Database (NHIRD), provided by Taiwan’s National Health Research Institutes. Taiwan’s NHIRD is population-based and derived from the claims data from the National Health Insurance program, a mandatory-enrollment, single-payment system that covers over 99 % of Taiwan’s population. The LHDB consists of a random sample of 120,000 de-identified diabetes incident cases from 1996 to 2013, who were tracked back to 1996 and followed up to 2013 to establish a longitudinal cohort. The LHDB is most representative of Taiwan’s diabetic population and provides the opportunity to conduct longitudinal studies to evaluate long-term outcomes of diabetes treatments.

### Study cohort

We identified patients aged ≥20 years with T2DM diagnosis (International Classification of Diseases, Ninth Revision, Clinical Modification, ICD-9-CM = 250.0X–250.9X, X = 0 or 2) from 1999–2013 LHDB. We further selected cases with any antidiabetic drug exposure during 2009–2010. The first claim date of an antidiabetic drug prescribed in 2009–2010 was defined as the index date. Patients who had any antidiabetic drugs before the index date were excluded, in order to include new users of antidiabetic drugs. The observation for each case started from the index date to the date of death or the end of 2013. The maximum follow-up time was 5 years (2009–2013) and the minimum was 3 years (2011–2013). The primary outcome of interest was major adverse cardiovascular events (MACEs) (a composite of CVD events including hospitalizations for ischemic stroke [ICD-9 codes 430–438], MI [ICD-9 codes 410, 412], and HF [ICD-9 codes 428]) and individual components of CVD. The secondary outcome was a hospitalization for hypoglycemia (ICD-9 codes: 250.8, 251.0, 251.1, and 251.2).

### Exposure to antidiabetic drugs

Medication utilization was identified using drug_no in the NHIRD and linked to the Anatomical Therapeutic Chemical (ATC) Classification System used to classify active ingredients of antidiabetic drugs: metformin, SU, DPP4i, pioglitazone, rosiglitazone, meglitinides, acarbose, and insulin. Patients were considered as unexposed to antidiabetic drugs (no antidiabetic drug exposure) if there was a gap of 30 days or more between two consecutive antidiabetic drug refills (“grace period”).

### Statistics

Population characteristics were analyzed using descriptive statistics, including means, standard deviation, frequency, and proportion. The crude incidence rate of CVD was calculated as the total number of CVD events during the follow-up period divided by person-years at risk. The person-years at risk was defined as the sum of patients from the index date (the first antidiabetic drug claim) to the diagnosis of the first CVD event, death, or the end of 2013, whichever came first. The time-varying Cox proportional hazards model was applied to evaluate the time to event for the effect of exposure to antidiabetic agents, adjusted for patients’ baseline characteristics: age, gender, comorbidity from 1 year prior to the index date (via Charlson comorbidity index; CCI [22]), diabetic complications (via adapted Diabetes Complication Severity Index; aDCSI [23–25]), CVD history, and co-medications for CVD, including α-blockers, β-blockers, angiotensin-converting enzyme inhibitors, angiotensin receptor blockers, calcium channel blockers, anti-platelet agents, anti-coagulants, diuretics, digoxin, and nitroglycerin.

The aforementioned baseline characteristics were treated as fixed effects in the Cox model, while antidiabetic drug exposure in the follow-up period was the random effect. Time-varying exposure to antidiabetic drugs was based on the expected duration of each prescription by using the “days supplied” field in the NHIRD. We first analyzed the outcomes of various antidiabetic drugs (i.e., DPP4i vs. non-DPP4i exposure, which included metformin, SU, pioglitazone, rosiglitazone, meglitinides, acarbose, and insulin, and non-antidiabetic drug exposure). Second, we assessed the comparative outcomes of antidiabetic drugs, where DPP4i served as the reference for comparison with other antidiabetic drugs. We attributed outcome events to the drugs the patient was expected to be receiving at the time of the event. We assumed no legacy or carryover effects of remote exposure to any of the antidiabetic drugs. Subgroup analyses were stratified by patients’ CVD history. Hazard ratios (HRs) and associated 95 % confidence intervals (CIs) adjusted for cluster variance were computed. The significance level of this study was set at 0.05. SAS software (version 9.4) was utilized for the aforementioned analyses.

## Results

**Fig. 1:**
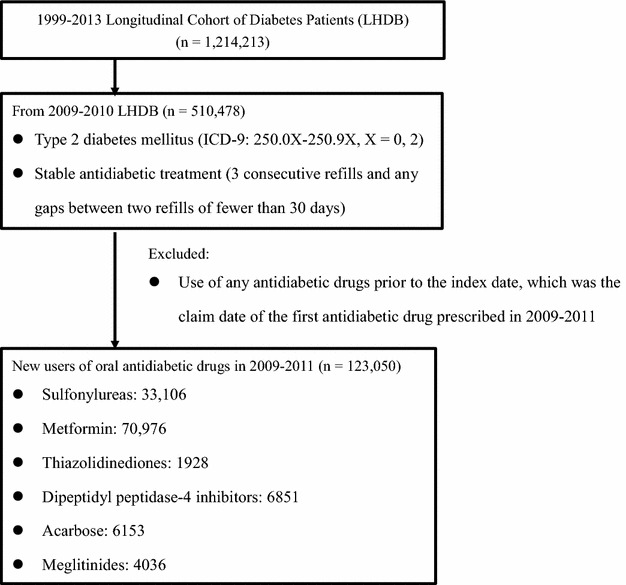
Flow diagram of the selection of study population

A total of 123,050 new users of oral antidiabetic drugs were included (Fig. 1), where metformin was the most commonly prescribed antidiabetic drug, and followed for a total of 362,656 person-years.

Table 1 shows the patients’ characteristics according to antidiabetic drug exposure at any point during the study. We identified 28,508 (8.1 %) patients who received DPP4i. Meglitinides users were relatively older and had more comorbidities and diabetic complications as compared to those exposed to other antidiabetic drugs.

**Table 1:** Patients’ characteristics according to antidiabetic drug exposure at any point during the study

	Exposed to metformin	Exposed to sulfonylureas	Exposed to pioglitazone	Exposed to rosiglitzone	Exposed to acarbose	Exposed to meglitinide	Exposed to DPP4i	Exposed to insulin
n	172,813	101,166	6831	1018	22,800	13,103	28,508	9632
Age	57.3 ± 13.2	58.0 ± 13.2	56.3 ± 12.6	55.8 ± 13.5	58.4 ± 13.8	61.6 ± 14.5	56.6 ± 13.2	60.9 ± 15.3
Sex (male), %	51.86	54.20	52.82	59.30	51.98	53.92	54.07	56.34
CCI (1–33)	4.3 ± 3.0	4.2 ± 3.1	4.1 ± 2.9	4.3 ± 3.1	4.7 ± 3.2	5.1 ± 3.4	4.4 ± 3.0	4.9 ± 3.5
aDCSI (0–13)	1.7 ± 2.3	1.8 ± 2.5	1.7 ± 2.4	2.1 ± 2.9	2.2 ± 2.9	2.8 ± 3.5	2.1 ± 2.8	2.6 ± 3.4
*Comorbidity history*								
Hypertension (%)	63.67	63.14	62.88	60.11	65.82	68.67	63.89	66.01
Dyslipidemia (%)	55.70	52.66	56.68	53.13	53.90	58.13	56.94	59.06
CAD (%)	31.68	30.72	30.26	29.16	35.01	38.13	32.07	35.22
Heart failure (%)	8.48	8.89	7.23	7.51	10.54	14.85	9.46	14.70
Stroke (%)	17.74	17.51	14.33	18.34	19.57	25.06	17.38	25.13
*Medication history*								
α-Blockers (%)	3.49	3.66	3.00	3.22	4.11	5.16	3.68	4.62
β-Blockers (%)	20.05	19.56	18.83	18.78	20.82	20.22	20.68	18.66
Diuretics (%)	16.57	16.96	15.59	13.15	17.72	19.99	14.67	19.36
CCB (%)	35.64	35.73	36.01	29.52	36.09	37.87	34.01	35.42
AECI/ARB (%)	32.60	31.48	34.48	35.06	36.20	35.29	38.46	32.82
Lipid lowering agent (%)	33.93	31.64	38.72	32.65	35.69	29.60	37.11	25.92
Anti-platelet (%)	24.53	23.84	23.35	23.43	28.05	30.75	26.56	28.98
NTG (%)	4.06	3.86	3.38	4.29	5.17	5.52	5.16	5.30
Anti-coagulants (%)	0.76	0.87	0.54	0.98	1.06	1.36	1.01	1.56
Digoxin (%)	1.82	2.00	1.52	2.42	2.33	3.59	2.05	3.60

*DPP4i* dipeptidyl peptidase-4 inhibitors, *CCI* Charlson Comorbidity Index, *aDCSI* adapted diabetes complication severity index, *CAD* coronary artery diseases, *CCB* calcium channel blockers, *ACEI/ARB* angiotensin-II-converting enzyme inhibitors/angiotensin receptor blockers, *NTG* nitroglycerin

Table 2 shows CVD risks for each antidiabetic drug as compared with non-exposure to a given antidiabetic drug (e.g., DPP4i users vs. non-DPP4i users). DPP4i, SU, acarbose, metformin, and pioglitazone users had significantly lower CVD risks than those of their counterparties (non-exposure to these drugs), while meglitinides and insulin users had significantly higher CVD risks as compared with those of patients without exposure to these drugs. There was no statistical difference in CVD risks between rosiglitazone users and non-rosiglitazone users.

**Table 2:** Hazard ratios of major adverse cardiovascular events (MACEs) associated with exposure to various antidiabetic drugs

MACEs	Time at risk (person-years)	Incidence rate (per 1000 person-years)	Unadjusted HR (95 % CI)	Adjusted HR* (95 % CI)	*p* value
DPP4i	28,508	39.56	0.82	0.83 (0.76, 0.91)	<0.0001
(ref.=non-DPP4i users)	334,148	41.53	–	–	–
Sulfonylureas	110,618	35.99	0.67	0.80 (0.77, 0.84)	<0.0001
(ref. = non-sulfonylureas users)	252,038	43.74	–	–	–
Acarbose	22,800	48.37	1.01	0.92 (0.85, 0.99)	0.0463
(ref. = non-acarbose users)	339,856	26.10	–	–	–
Meglitinides	13,103	102.49	2.23	1.46 (1.35, 1.58)	<0.0001
(ref. = non-meglitinide users)	349,553	39.08	–	–	–
Insulin	9632	206.06	4.75	3.53 (3.23, 3.87)	<0.0001
(ref. = non-insulin users)	353,024	36.88	–	–	–
Metformin	172,813	30.87	0.48	0.66 (0.63, 0.69)	<0.0001
(ref. = non-metformin users)	189,843	45.00	–	–	–
Pioglitazone	4678	24.15	0.49	0.61 (0.50, 0.75)	<0.0001
(ref. = non-pioglitazone users)	357,978	41.60	–	–	–
Rosiglitazone	504	35.64	0.69	0.78 (0.49, 1.26)	0.3187
(ref. = non-rosiglitazone users)	362,152	41.38	–	–	–

*HR* hazard ratio, *CI* confidence interval, *DPP4i* dipeptidyl peptidase-4 inhibitors

* Adjusted hazard ratios were estimated from the Cox models adjusted for age, sex, diabetes duration, comorbidity history (hypertension, hyperlipidemia, coronary artery diseases, stroke, myocardial infarction, heart failure, Charlson comorbidity index), diabetic complications (via adapted diabetic complication severity index), co-medications (α-blockers, β-blockers, diuretics, calcium channel blockers, angiotensin-II-converting enzyme inhibitors/angiotensin receptor blockers, lipid-lowering agents, anti-platelet agents/anticoagulants, nitroglycerin, digoxin)

Table 3 shows the comparative CVD and hypoglycemic risks of antidiabetic drugs, where DPP4i served as the reference. DPP4i users had a significantly lower risk for MACEs than that of meglitinides and insulin users, but higher than that of metformin and pioglitazone users. Also, DPP4i users had a significantly lower stroke risk than that of meglitinides and insulin users, but higher than that of pioglitazone users. DPP4i users had a significantly lower MI risk than that of meglitinides and insulin users, but higher than that of metformin users. DPP4i users had a significantly lower HF risk than that of meglitinides and insulin users, but higher than that of SU and metformin users. DPP4i users had a significantly lower hypoglycemic risk than that of meglitinides and insulin users, but higher than that of metformin users. Subgroup analyses for the patients with and without CVD history (Tables 4, 5) show trends similar to those from the primary analysis (Table 3).

**Table 3:** Hazard ratios of cardiovascular diseases and hypoglycemic events associated with various antidiabetic drugs, as compared to DPP4i as reference

	Time at risk (person-years)	Incidence rate (per 1000 person-years)	Unadjusted HR (95 % CI)	Adjusted HR* (95 % CI)	*p* value
*MACEs*					
Antidiabetic drug (ref. = DPP4i)	28,508	39.56	–	–	–
No antidiabetic drug	101,166	62.02	1.52 (1.39, 1.65)	1.31 (1.20, 1.43)	<0.0001
Sulfonylureas	110,618	35.99	0.89 (0.81, 0.96)	0.96 (0.88, 1.05)	0.3942
Acarbose	22,800	48.37	1.20 (1.08, 1.33)	1.07 (0.96, 1.18)	0.2098
Meglitinides	13,103	102.49	2.52 (2.27, 2.78)	*1.68 (1.52, 1.86)*	*<0.0001*
Insulin	9632	206.06	5.08 (4.60, 5.61)	*3.73 (3.35, 4.14)*	*<0.0001*
Metformin	172,813	30.87	0.76 (0.69, 0.82)	*0.87 (0.79, 0.94)*	*0.0009*
Pioglitazone	4678	24.15	0.59 (0.47, 0.73)	*0.70 (0.56, 0.86)*	*0.0011*
Rosiglitazone	504	35.64	0.78 (0.48, 1.23)	0.89 (0.55, 1.42)	0.6296
*Ischemic stroke*					
Antidiabetic drug (ref. = DPP4i)	28,508	20.69	–	–	–
No antidiabetic drug	101,166	35.83	1.68 (1.49, 1.89)	1.42 (1.26, 1.60)	<0.0001
Sulfonylureas	110,618	21.95	1.03 (0.92, 1.16)	1.06 (0.94, 1.19)	0.3465
Acarbose	22,800	26.18	1.24 (1.08, 1.43)	1.09 (0.94, 1.25)	0.2432
Meglitinides	13,103	50.98	2.39 (2.07, 2.75)	*1.58 (1.37, 1.81)*	*<0.0001*
Insulin	9632	111.07	5.23 (4.58, 5.97)	*3.99 (3.47, 4.56)*	*<0.0001*
Metformin	172,813	20.61	0.97 (0.86, 1.08)	1.03 (0.92, 1.15)	0.5630
Pioglitazone	4678	13.25	0.62 (0.47, 0.82)	*0.71 (0.53, 0.94)*	*0.0166*
Rosiglitazone	504	23.76	0.99 (0.55, 1.75)	1.06 (0.59, 1.87)	0.8551
*Myocardial infarction*					
Antidiabetic drug (ref. = DPP4i)	28,508	5.19	–	–	–
No antidiabetic drug	101,166	7.01	1.47 (1.20, 1.80)	1.45 (1.18, 1.78)	0.0003
Sulfonylureas	110,618	4.21	0.83 (0.68, 1.01)	0.98 (0.80, 1.78)	0.8257
Acarbose	22,800	5.08	1.00 (0.77, 1.29)	0.98 (0.75, 1.27)	0.8993
Meglitinides	13,103	9.53	1.89 (1.49, 2.39)	*1.49 (1.17, 1.90)*	*0.0011*
Insulin	9632	17.64	3.43 (2.72, 4.33)	*2.77 (2.17, 3.51)*	*<0.0001*
Metformin	172,813	3.30	0.66 (0.54, 0.79)	*0.80 (0.50, 1.39)*	*0.0234*
Pioglitazone	4678	3.63	0.70 (0.42, 1.16)	0.84 (0.50, 1.39)	0.5007
Rosiglitazone	504	3.96	0.81 (0.20, 3.23)	0.92 (0.23, 3.63)	0.9037
*Heart failure*					
Antidiabetic drug (ref. = DPP4i)	28,508	15.92	–	–	–
No antidiabetic drug	101,166	25.23	1.50 (1.30, 1.71)	1.28 (1.11, 1.47)	0.0004
Sulfonylureas	110,618	11.78	0.71 (0.62, 0.81)	*0.86 (0.74, 1.08)*	*0.0322*
Acarbose	22,800	19.82	1.21 (1.03, 1.42)	1.08 (0.92, 1.26)	0.3227
Meglitinides	13,103	48.23	2.91 (2.48, 3.40)	*1.85 (1.58, 2.16)*	*<0.0001*
Insulin	9632	92.80	5.67 (4.87, 6.58)	*3.71 (3.14, 4.37)*	*<0.0001*
Metformin	172,813	8.51	0.51 (0.44, 0.59)	*0.66 (0.57, 0.75)*	*<0.0001*
Pioglitazone	4678	9.40	0.57 (0.39, 0.80)	0.76 (0.53, 1.07)	0.1229
Rosiglitazone	504	7.92	0.42 (0.15, 1.13)	0.53 (0.19, 1.46)	0.2226
*Hypoglycemia*					
Antidiabetic drug (ref. = DPP4i)	28,508	5.82	–	–	–
No antidiabetic drug	101,166	5.23	0.83 (0.67, 1.00)	0.76 (0.62, 0.92)	0.0053
Sulfonylureas	110,618	6.21	1.00 (0.84, 1.19)	1.08 (0.91, 1.29)	0.3621
Acarbose	22,800	5.83	0.96 (0.77, 1.19)	0.90 (0.72, 1.11)	0.3402
Meglitinides	13,103	18.16	2.91 (2.35, 3.59)	*2.09 (1.68, 2.58)*	*<0.0001*
Insulin	9632	39.44	6.41 (5.24, 7.84)	*4.55 (3.67, 5.64)*	*<0.0001*
Metformin	172,813	2.69	0.43 (0.35, 0.52)	*0.50 (0.41, 0.60)*	*<0.0001*
Pioglitazone	4678	5.77	0.93 (0.62, 1.40)	1.07 (0.71, 1.60)	0.7471
Rosiglitazone	504	11.88	1.50 (0.66, 3.37)	1.58 (0.70, 3.55)	0.2679

The results in italics above indicate significant findings in the Cox models

*HR* hazard ratio, *CI* confidence interval, *MACEs* major adverse cardiovascular events, *DPP4i* dipeptidyl peptidase-4 inhibitors

* Adjusted hazard ratios were estimated from the Cox models adjusted for age, sex, diabetes duration, comorbidity history (hypertension, hyperlipidemia, coronary artery diseases, stroke, myocardial infarction, heart failure, Charlson comorbidity index), diabetic complications (via adapted diabetic complication severity index), co-medications (α-blockers, β-blockers, diuretics, calcium channel blockers, angiotensin-II-converting enzyme inhibitors/angiotensin receptor blockers, lipid-lowering agents, anti-platelet agents/anticoagulants, nitroglycerin, digoxin)

**Table 4:** Subgroup analysis of hazard ratios of cardiovascular diseases and hypoglycemic events associated with various antidiabetic drugs, as compared to DPP4i as reference, in the patients with CVD history

	Time at risk (person-years)	Incidence rate (per 1000 person-years)	Unadjusted HR (95 % CI)	Adjusted HR* (95 % CI)
*MACEs*				
Antidiabetic drug (ref. = DPP4i)	11,532	71.79	–	–
No antidiabetic drug	43,967	109.05	1.47 (1.32, 1.62)	1.27 (1.14, 1.40)
Sulfonylureas	40,852	67.48	0.92 (0.82, 1.01)	0.97 (0.87, 1.08)
Acarbose	9972	80.52	1.10 (0.97, 1.24)	1.01 (0.89, 1.14)
Meglitinides	6346	160.39	2.16 (1.91, 2.44)	1.54 (1.36, 1.73)
Insulin	3919	362.57	4.90 (0.68, 0.84)	3.30 (2.90, 3.75)
Metformin	67,237	56.33	0.76 (0.51, 0.83)	0.88 (0.78, 0.97)
Pioglitazone	1683	48.72	0.65 (0.51, 0.83)	0.74 (0.57, 0.95)
Rosiglitazone	186	75.00	0.91 (0.53, 1.54)	0.96 (0.56, 1.64)
*Ischemic stroke*				
Antidiabetic drug (ref. = DPP4i)	11,532	37.02	–	–
No antidiabetic drug	43,967	61.47	1.61 (1.39, 1.85)	1.38 (1.19, 1.59)
Sulfonylureas	40,852	40.19	1.06 (0.91, 1.22)	1.07 (0.92, 1.23)
Acarbose	9972	41.51	1.10 (0.92, 1.29)	1.01 (0.85,1.18)
Meglitinides	6346	79.40	2.08 (1.75, 2.45)	1.48 (1.24, 1.74)
Insulin	3919	183.96	4.82 (4.10, 5.65)	3.37 (2.86, 3.96)
Metformin	67,237	36.78	0.97 (0.84, 1.10)	1.05 (0.91, 1.20)
Pioglitazone	1683	23.17	0.60 (0.43, 0.84)	0.67 (0.47, 0.94)
Rosiglitazone	186	42.85	1.01 (0.50, 2.02)	0.99 (0.49, 2.00)
*Myocardial infarction*				
Antidiabetic drug (ref. = DPP4i)	11,532	8.67	–	–
No antidiabetic drug	43,967	11.05	1.38 (1.08, 1.76)	1.34 (1.04, 1.71)
Sulfonylureas	40,852	7.31	0.87 (0.68, 1.10)	0.99 (0.72, 1.26)
Acarbose	9972	7.82	0.92 (0.67, 1.26)	0.91 (0.66, 1.25)
Meglitinides	6346	13.86	1.65 (1.23, 2.20)	1.32 (0.98, 1.77)
Insulin	3919	27.81	3.25 (2.45, 4.30)	2.41 (1.80, 3.21)
Metformin	67,237	5.35	0.64 (0.50, 0.80)	0.78 (0.61, 0.98)
Pioglitazone	1683	7.12	0.83 (0.45, 1.52)	0.93 (0.50, 1.71)
Rosiglitazone	186	10.71	1.32 (0.33, 5.32)	1.46 (0.36, 5.81)
*Heart failure*				
Antidiabetic drug (ref. = DPP4i)	11,532	30.26	–	–
No antidiabetic drug	43,967	48.71	1.52 (1.29, 1.78)	1.28 (1.08, 1.49)
Sulfonylureas	40,852	24.03	0.77 (0.65, 0.90)	0.86 (0.72, 1.02)
Acarbose	9972	36.19	1.17 (0.97, 1.39)	1.07 (0.89, 1.28)
Meglitinides	6346	77.36	2.45 (2.03, 2.95)	1.66 (1.38, 1.98)
Insulin	3919	178.60	5.72 (4.78, 6.83)	3.52 (2.88, 4.28)
Metformin	67,237	17.20	0.55 (0.46, 0.64)	0.68 (0.57, 0.80)
Pioglitazone	1683	23.17	0.73 (0.50, 1.07)	0.89 (0.59, 1.31)
Rosiglitazone	186	21.42	0.61 (0.22, 1.63)	0.72 (0.26, 1.97)
*Hypoglycemia*				
Antidiabetic drug (ref. = DPP4i)	11,532	7.37	–	–
No antidiabetic drug	43,967	7.32	0.92 (0.70, 1.20)	0.83 (0.63, 1.07)
Sulfonylureas	40,852	10.13	1.31 (1.02, 1.67)	1.44 (1.12, 1.82)
Acarbose	9972	7.52	0.98 (0.72, 1.32)	0.92 (0.68, 1.24)
Meglitinides	6346	24.10	3.09 (2.35, 4.03)	2.20 (1.68, 2.88)
Insulin	3919	45.67	5.91 (4.50, 7.74)	3.73 (2.77, 5.01)
Metformin	67,237	3.56	0.46 (0.35, 0.59)	0.54 (0.42, 0.70)
Pioglitazone	1683	8.31	1.08 (0.61, 1.90)	1.25 (0.70, 2.20)
Rosiglitazone	186	21.42	2.31 (0.84, 6.32)	2.42 (0.88, 6.65)

*CVD* cardiovascular disease, *HR* hazard ratio, *CI* confidence interval, *MACEs* major adverse cardiovascular events, *DPP-4 inhibitors* dipeptidyl peptidase-4 inhibitors

* Adjusted hazard ratios were estimated from the Cox models adjusted for age, sex, diabetes duration, comorbidity history (hypertension, hyperlipidemia, coronary artery diseases, stroke, myocardial infarction, heart failure, Charlson comorbidity index), diabetic complications (via adapted diabetic complication severity index), co-medications (α-blockers, β-blockers, diuretics, calcium channel blockers, angiotensin-II-converting enzyme inhibitors/angiotensin receptor blockers, lipid-lowering agents, anti-platelet agents/anticoagulants, nitroglycerin, digoxin)

**Table 5:** Subgroup analysis of hazard ratios of cardiovascular diseases and hypoglycemic events associated with various antidiabetic drugs, as compared to DPP4i as reference, in the patients without CVD history

	Time at risk (person-years)	Incidence rate (per 1000 person-years)	Unadjusted HR (95 % CI)	Adjusted HR* (95 % CI)
*MACEs*				
Antidiabetic drug (ref. = DPP4i)	16,975	17.67	–	–
No antidiabetic drug	57,199	25.84	1.50 (1.29, 1.74)	1.38 (1.19, 1.60)
Sulfonylureas	69,766	17.55	0.99 (0.86, 1.15)	0.93 (0.80, 1.07)
Acarbose	12,828	23.38	1.33 (1.10, 1.60)	1.22 (1.01, 1.47)
Meglitinides	6756	48.10	2.73 (2.28, 3.26)	2.04(1.70, 2.44)
Insulin	5713	98.70	5.56 (4.70, 6.58)	5.48 (4.60, 6.50)
Metformin	105,576	14.65	0.83 (0.72, 0.96)	0.82 (0.70, 0.94)
Pioglitazone	2995	10.35	0.58 (0.39, 0.87)	0.61 (0.40, 0.90)
Rosiglitazone	318	12.56	0.68 (0.25, 1.83)	0.68 (0.25, 1.84)
*Ischemic stroke*				
Antidiabetic drug (ref. = DPP4i)	16,975	9.60	–	–
No antidiabetic drug	57,199	16.11	1.70 (1.38, 2.07)	1.52 (1.23, 1.86)
Sulfonylureas	69,766	11.28	1.17 (0.96, 1.42)	1.04 (0.85, 1.27)
Acarbose	12,828	14.26	1.49 (1.15, 1.92)	1.34 (1.03, 1.74)
Meglitinides	6756	24.27	2.52 (1.96, 3.22)	1.85 (1.44, 2.36)
Insulin	5713	61.08	6.32 (5.02, 7.94)	6.56 (5.21, 8.27)
Metformin	105,576	10.32	1.07 (0.88, 1.29)	1.01 (0.82, 1.22)
Pioglitazone	2995	7.67	0.80 (0.49, 1.29)	0.81 (0.50, 1.32)
Rosiglitazone	318	12.56	1.21 (0.44, 3.27)	1.22 (0.44, 3.32)
*Myocardial infarction*				
Antidiabetic drug (ref. = DPP4i)	16,975	2.82	–	–
No antidiabetic drug	57,199	3.91	1.55 (1.09, 2.20)	1.56 (1.09, 2.22)
Sulfonylureas	69,766	2.39	0.88 (0.63, 1.22)	0.93 (0.66, 1.29)
Acarbose	12,828	2.96	1.08 (0.68, 1.70)	1.09 (0.69, 1.71)
Meglitinides	6756	5.47	2.02 (1.33, 3.04)	1.78 (1.17, 2.69)
Insulin	5713	10.67	3.84 (2.54, 5.79)	3.38 (2.23, 5.10)
Metformin	105,576	1.99	0.74 (0.53, 1.02)	0.81 (0.58, 1.13)
Pioglitazone	2995	1.66	0.59 (0.23, 1.49)	0.63 (0.24, 1.60)
Rosiglitazone	318	0	0	0
*Heart failure*				
Antidiabetic drug (ref. = DPP4i)	16,975	6.18	–	–
No antidiabetic drug	57,199	7.18	1.19 (0.92, 1.52)	1.13 (0.87, 1.45)
Sulfonylureas	69,766	4.61	0.75 (0.58, 0.95)	0.73 (0.57, 0.94)
Acarbose	12,828	7.09	1.15 (0.84, 1.56)	1.06 (0.78, 1.44)
Meglitinides	6756	20.86	3.38 (2.55, 4.46)	2.45 (1.85, 3.22)
Insulin	5713	33.95	5.45 (4.17, 7.10)	4.75(3.54, 6.35)
Metformin	105,576	2.97	0.48 (0.37, 0.61)	0.51 (0.39, 0.65)
Pioglitazone	2995	1.66	0.27 (0.10, 0.66)	0.29 (0.11, 0.71)
Rosiglitazone	318	0	0	0
*Hypoglycemia*				
Antidiabetic drug (ref. = DPP4i)	16,975	4.77	–	–
No antidiabetic drug	57,199	3.63	0.69 (0.51, 0.92)	0.69 (0.51, 0.92)
Sulfonylureas	69,766	3.92	0.76 (0.59, 0.98)	0.78 (0.60, 1.00)
Acarbose	12,828	4.52	0.91 (0.65, 1.24)	0.89 (0.64, 1.22)
Meglitinides	6756	12.58	2.44 (1.70, 3.49)	1.93 (1.33, 2.77)
Insulin	5713	35.17	6.95 (5.17, 9.34)	5.47 (4.00, 7.48)
Metformin	105,576	2.13	0.41 (0.30, 0.55)	0.46 (0.34, 0.61)
Pioglitazone	2995	4.34	0.84 (0.47, 1.50)	0.93 (0.52, 1.65)
Rosiglitazone	318	6.28	0.89 (0.22, 3.57)	0.91 (0.23, 3.56)

*CVD* cardiovascular disease, *HR* hazard ratio, *CI* confidence interval, *MACEs* major adverse cardiovascular events, *DPP-4 inhibitors* dipeptidyl peptidase-4 inhibitors

* Adjusted hazard ratios were estimated from the Cox models adjusted for age, sex, diabetes duration, comorbidity history (hypertension, hyperlipidemia, coronary artery diseases, stroke, myocardial infarction, heart failure, Charlson comorbidity index), diabetic complications (via adapted diabetic complication severity index), co-medications (α-blockers, β-blockers, diuretics, calcium channel blockers, angiotensin-II-converting enzyme inhibitors/angiotensin receptor blockers, lipid-lowering agents, anti-platelet agents/anticoagulants, nitroglycerin, digoxin)

## Discussion

This was a large cohort study with a long-term follow-up to assess CVD and hypoglycemic risks of DPP4i as compare with other antidiabetic drugs. DPP4i users had significantly lower CVD risks as compared to non-DPP4i users. DPP4i users had significantly lower CVD risks than that for those treated with meglitinides and insulin, but not than that for those treated with metformin and pioglitazone.

## Comparison with previous studies

Several studies have evaluated CVD risks in DPP4i users as compared to those of non-DPP4i users, which are similar to the approach in the first part of our analysis (the results presented in Table 2). Eurich et al.’s study, based on commercially insured US claims, showed that sitagliptin users did not have increased risks for CVD-related hospitalizations as compared with those of non-sitagliptin users (0.90, 95 % CI 0.77–1.07) [26]. Kim et al. utilized US commercial insurance claims data and found that DPP4i users had significantly lower risks for composite CVD events (including stroke, MI, and HF) (0.87, 95 % CI 0.79–0.96), with similar trends observed in patients with CVD history [16]. Chen et al.’s study utilized Taiwan’s NHIRD 2009–2011 and found that DPP4i use in T2DM patients with stroke [27] or MI [28] history was not associated with increased CVD risks as compared with non-DPP4i users. Consistent with the aforementioned studies, the present research based on Taiwan’s NHIRD 1999–2013 found that DPP4i users did not have increased risks for CVD as compared with non-DPP4i users (Table 2).

## Comparative CVD risks of DPP4i with other antidiabetic drugs

We further performed comparative analysis of CVD risks of antidiabetic drugs. DPP4i users appeared to have lower CVD risks as compared with those of insulin and meglitinides users, but higher CVD risks than those of metformin and pioglitazone users. Eurich et al.’s study found that insulin users had significantly higher risks for CVD-related hospitalizations than those of non-insulin users (HR 2.15, 95 CI %: 1.85–2.51) [26]. Several possible reasons may explain the high CVD risks in insulin users. First, a harmful effect of insulin on the vascular endothelium has been suggested and increased insulin dosage appeared to be associated with increased CVD risks [29]. Second, hypoglycemia is a common side effect observed in insulin users. Hypoglycemia has been associated with increased CVD risks. The present study found that insulin-treated T2DM patients had 4.55 times higher hypoglycemic risk as compared to that of those treated with DPP4i (Table 3), which might in part explain higher CVD risks in insulin users.

Meglitinides have been related to variable degrees of undesirable CVD risks [30]. Repaglinide, a meglitinide that acts by closing ATP-dependent potassium channels, appears to be associated with a risk of adverse cardiovascular sequelae similar to that for SU [30]. Additionally, we found that meglitinides users appeared to be older and advanced diabetic patients, in terms of comorbidity and diabetic complications, as compared to those treated with other OHAs (i.e., DPP4i) (Table 1). And, meglitinides users had a significantly higher CVD risks as compared with non-meglitinides users (Table 2). Meglitinides users might thus have poor prognosis and higher CVD risks as compared to those on other OHAs such as DPP4i (Table 3).

Previous evidence showed that metformin users had significantly lower CVD risks as compared with non-metformin users, with a possible mechanism being the attenuation of atrial cell tachycardia-induced myolysis oxidative stress [31]. Limited research has compared the CVD risks of DPP4i users with those of metformin users. A recent clinical trial showed that, in terms of glycemic control, metformin monotherapy was superior to DPP4i monotherapy; however, no difference in CVD risks between the two treatment groups were noticed, in part due to the limited study period (i.e., 12 months) [32]. This observational study with a relatively long follow-up time showed that metformin appeared to be associated with significantly lower CVD risks as compared to DPP4i.

TZDs (i.e., rosiglitazone,pioglitazone) have been associated with increased risks for stroke, HF, and all-cause mortality [33–36], which appeared to be largest in the elderly (i.e., >60 years) and in patients treated with rosiglitazone [36]. Practice guidelines state that rosiglitazone and pioglitazone are not recommended for diabetic patients with pre-existing heart diseases or at risk for CVD (i.e., decreased ventricular function) [37]. Accordingly, the patients who receive TZDs are likely to be underlying at low CVD risks. Based on our cohort, we found that TZDs users had relatively lower hypertension, dyslipidemia, coronary artery diseases, HF, and stroke at baseline as compared to those of patients exposed to DPP4i (Table 1), which might explain lower CVD risks in TZDs-treated patients (e.g., pioglitazone) as compared with those of DPP4i users. In other words, there appears to be potential confounding by indication regarding TZDs use, which might influence our comparative analysis of CVD risks between TZDs and DPP4i. Although we adjusted for patients’ baseline comorbidities (i.e., CVD history) in the analysis and stratified the analysis by patients’ CVD history, other unmeasured biases (e.g., weight gain, diet, exercise, physicians’ behaviors) for CVD outcomes may still exist. We were thus unable to determine whether DPP4i use is associated with lower or higher CVD risks as compared to TZDs because potential confounding by indication could not be excluded.

A recent review of vitro studies and preliminary trials concluded potential cardiovascular benefits of alpha-glucosidase inhibitors (i.e., acarbose) in diabetic patients [38], particularly for those with IGT [8, 9]. Intervening on postprandial hyperglycemia, a key component of mechanisms linked to increased CVD incidences [39, 40], acarbose was associated with a favorable impact on CVD surrogate markers [41, 42]. Our analysis showed that acarbose use was associated with lower CVD risks as compared to non-acarbose use (Table 2). However, lack of previous research evaluated comparative CVD risks between DPP4i and alpha-glucosidase inhibitors. Although we found no significant difference in CVD risks between DPP4i and acarbose (Table 3), further study is needed to confirm our findings, especially for diabetic patients with IGT.

Today, most studies compared the CVD risks of DPP4i with SU as a group [17, 43, 44], not individual SU. However, individual SU appear to have different degrees of desirable or undesirable cardiovascular effects. Glimepiride, a third-generation SU, might provide potential cardiovascular benefits because of its favorable glycemic control, especially postprandial glucose lowering effects [45], anti-oxidative properties [46], and maintaining myocardial preconditioning [47]. In terms of reducing CVD risks, glimepiride or gliclazide with a specific influence on pancreatic ATP-sensitive K^+^ channels might be superior to glibenclamide [45], which blocks mitochondrial ATP-sensitive K^+^ channels in cardiac myocytes, resulting in the inhibition of ischemic preconditioning [48]. Also, a previous population-based cohort study showed that glipizide was associated with increased CVD risks as compared to other SU. Hence, further study is anticipated to assess whether pharmacological differences between individual drugs translate into differences in their associated CVD risks. Our additional analyses showed that, as compared to glibenclamide, gliclazide had a significantly lower risk for MACEs, and gliclazide and glimepiride had a significantly lower risk for stroke (Additional file 1: Table S1). In this regard, we further compared the CVD risks of DPP4i with individual SU. The results showed that, as compared to DPP4i, glipizide and glibenclamide had significantly higher risks for MACEs and stroke (Additional file 1: Table S2).

## Study limitations

First, laboratory data (e.g., HbA1c) were not available in the NHIRD claims data. However, we used surrogat indicators to adjust for patients’ baseline diabetes severity, including aDCSI and diabetes duration. Second, because of the nature of an observational study, potential confounding by indication could not be eliminated. Also, potential residual confounding by incomplete adjustment for unmeasured biases (i.e., lifestyle risk factors, physicians’ preferences and behaviors) for study outcomes may exist. Third, another class of incretin drug, namely GLP-1 receptor agonists (GLP-1 RA), was not analyzed. GLP-1 RA was introduced to Taiwan’s national formulary in 2013. Since our data were only available up to the end of 2013, GLP-1 RA users only accounted for a small proportion of our study population. Fourth, potential misclassification may exist when defining CVD events based on Taiwan’s NHIRD. However, previous validation studies for the identification of CVD events (i.e., MI [49], stroke [50]) from the NHIRD showed high sensitivity and positive predictive values. Fifth, individual DPP4i might be associated with variable degrees of desirable or undesirable cardiovascular outcomes [51]. The SAVOR-TIMI 53 trial reported an increased HF risk in saxagliptin-treated patients [12], which was not seen with other DPP4i. The present study analyzed individual DPP4i as a group because our preliminary analyses showed no significant difference in comparative risks for MACEs and HF of individual DPP4i (sitagliptin, vildagliptin, saxagliptin, linagliptin) (Additional file 1: Table S3). Further study is anticipated to clarify the mechanisms underlying the difference in CVD risks among individual DPP4i. Lastly, our results might only be generalizable to a Chinese population under universal healthcare insurance coverage.

## Conclusions

Understanding comparative effects of antidiabetic drugs provides a basis for guiding clinical care for T2DM patients. The present study shows that the use of DPP4i was not associated with increased CVD risks and that DPP4i-treated patients appeared to have lower CVD risks as compared with non-DPP4i users, except metformin users.

## Additional file

10.1186/s12933-016-0350-4 Hazards ratios of cardiovascular diseases as compared individual sulfonylureas with glibenclamide as reference. **Table S2.** Hazards ratios of cardiovascular diseases as compared individual sulfonyureas with DPP4i as reference. **Table S3.** Hazards ratios of cardiovascular diseases as compared individual DPP4i with sitagliptin as reference.

## Abbreviations

aDCSI: adapted Diabetes Complication Severity Index
ATC: Anatomical Therapeutic Chemical
CCI: Charlson comorbidity index
CIs: confidence intervals
CVD: cardiovascular diseases
DPP4i: dipeptidyl peptidase-4 inhibitors
GLP-1 RA: GLP-1 receptor agonists
HF: heart failure
HRs: hazard ratios
ICD-9-CM: International Classification of Diseases, Ninth Revision, Clinical Modification
IGT: impaired glucose tolerance
LHDB: Longitudinal Cohort of Diabetes Patients database
MACEs: major adverse cardiovascular events
MI: myocardial infarction
NHIRD: National Health Insurance Research Database
OHAs: oral hypoglycemic agents
SU: sulfonylureas
TZDs: thiazolidinediones
