# Supplementary material for: Risks of cardiovascular diseases associated with dipeptidyl peptidase-4 inhibitors and other antidiabetic drugs in patients with type 2 diabetes: a nation-wide longitudinal study
Source: Cardiovasc Diabetol. 2016 Mar 1;15:41. doi: 10.1186/s12933-016-0350-4 (PMC4774127; doi:10.1186/s12933-016-0350-4)
Supplement: Supplementary file 1 — 10.1186/s12933-016-0350-4 Hazards ratios of cardiovascular diseases as compared individual sulfonylureas with glibenclamide as reference. Table S2. Hazards ratios of cardiovascular diseases as compared individual sulfonyureas with DPP4i as reference. Table S3. Hazards ratios of cardiovascular diseases as compared individual DPP4i with sitagliptin as reference. [file 12933_2016_350_MOESM1_ESM.docx]

**Table S1: Hazards ratios of cardiovascular diseases as compared individual sulfonylureas* with glibenclamide as reference**

| **MACEs**  **(glibenclamide as reference)** | **Unadjusted HR** (95% CI) | **Adjusted HR**** (95% CI) | *p* value |
| --- | --- | --- | --- |
| glipizide | 1.53 (1.31, 1.80) | 1.15 (0.97, 1.35) | 0.1059 |
| gliclazide | 0.79 (0.68, 0.91) | 0.81 (0.69, 0.94) | 0.0045 |
| glimepiride | 0.87 (0.76, 0.99) | 0.90 (0.79, 1.02) | 0.0959 |
| **Ischemic stroke**  **(glibenclamide as reference)** | **Unadjusted HR** (95% CI) | **Adjusted HR**** (95% CI) | *p* value |
| glipizide | 1.51 (1.24, 1.85) | 1.16 (0.94, 1.41) | 0.1621 |
| gliclazide | 0.75 (0.62, 0.91) | 0.76 (0.63, 0.92) | 0.0045 |
| glimepiride | 0.81 (0.69, 0.95) | 0.83 (0.71, 0.97) | 0.0203 |
| **Myocardial infarction (glibenclamide as reference)** | **Unadjusted HR** (95% CI) | **Adjusted HR**** (95% CI) | *p* value |
| glipizide | 1.40 (0.90, 2.16) | 1.07 (0.68, 1.67) | 0.7725 |
| gliclazide | 0.99 (0.69, 1.44) | 0.99 (0.68, 1.43) | 0.9456 |
| glimepiride | 1.00 (0.711, 1.41) | 0.98 (0.69, 1.38) | 0.9041 |
| **Heart failure**  **(glibenclamide as reference)** | **Unadjusted HR** (95% CI) | **Adjusted HR**** (95% CI) | *p* value |
| glipizide | 1.61 (1.20, 2.15) | 1.13 (0.83, 1.53) | 0.4379 |
| gliclazide | 0.79 (0.61, 1.03) | 0.86 (0.65, 1.12) | 0.2557 |
| glimepiride | 1.01 (0.80, 1.28) | 1.09 (0.85, 1.38) | 0.5049 |

Abbreviations: HR: hazard ratio, CI: confidence interval, MACEs: major adverse cardiovascular events (a composite of cardiovascular disease events including hospitalizations for ischemic stroke, myocardial infarction, and heart failure), DPP4i: dipeptidyl peptidase-4 inhibitors.

* The analyses did not include chlorpropamide and tolazamide because they are the 1st generation of SU and were not used very frequently; they accounted less 1% of total sulfonyurea prescriptions in our study cohort. Also, gliquidone was not included in analysis because it was not used very frequently either (they accounted less 1% of total sulfonyurea prescriptions).

** Adjusted hazard ratios were estimated from the Cox models adjusted for age, sex, diabetes duration, comorbidity history (hypertension, hyperlipidemia, coronary artery diseases, stroke, myocardial infarction, heart failure, Charlson comorbidity index), diabetic complications (via adapted diabetic complication severity index), co-medications ($\alpha-$blockers, $\beta-$blockers, diuretics, calcium channel blockers, angiotensin-II-converting enzyme inhibitors/angiotensin receptor blockers, lipid-lowering agents, anti-platelet agents/anticoagulants, nitroglycerin, digoxin).

**Table S2: Hazards ratios of cardiovascular diseases as compared individual sulfonyureas with DPP4i as reference**

| **MACEs**  **(DPP4i as reference)** | **Unadjusted HR**  (95% CI) | **Adjusted HR***  (95% CI) | *p* value |
| --- | --- | --- | --- |
| Glibenclamide | 0.93 (0.81, 1.08) | 1.19 (1.03, 1.38) | 0.0222 |
| Glipizide | 1.43 (1.25, 1.63) | 1.38 (1.20, 1.59) | <.0001 |
| Gliclazide | 0.73 (0.65, 0.83) | 0.96 (0.85, 1.09) | 0.5426 |
| Glimepiride | 0.81 (0.74, 0.89) | 1.07 (0.96, 1.18) | 0.2091 |
| **Ischemic stroke**  **(DPP4i as reference)** | **Unadjusted HR**  (95% CI) | **Adjusted HR***  (95% CI) | *p* value |
| Glibenclamide | 1.15 (0.96, 1.37) | 1.35 (1.12, 1.63) | 0.0015 |
| Glipizide | 1.74 (1.46, 2.07) | 1.57 (1.31, 1.89) | <.0001 |
| Gliclazide | 0.87 (0.73, 1.03) | 1.03 (0.86, 1.23) | 0.7611 |
| Glimepiride | 0.93 (0.82, 1.06) | 1.12 (0.98, 1.28) | 0.1085 |
| **Myocardial infarction (DPP4i as reference)** | **Unadjusted HR**  (95% CI) | **Adjusted HR***  (95% CI) | *p* value |
| Glibenclamide | 0.76 (0.53, 1.10) | 1.04 (0.71, 1.52) | 0.8462 |
| Glipizide | 1.06 (0.75, 1.50) | 1.13 (0.79, 1.63) | 0.5040 |
| Gliclazide | 0.76 (0.58, 0.98) | 1.03 (0.78, 1.36) | 0.8482 |
| Glimepiride | 0.76 (0.61, 0.94) | 1.02 (0.81, 1.28) | 0.8611 |
| **Heart failure**  **(DPP4i as reference)** | **Unadjusted HR**  (95% CI) | **Adjusted HR***  (95% CI) | *p* value |
| Glibenclamide | 0.67 (0.52, 0.87) | 0.99 (0.76, 1.29) | 0.9539 |
| Glipizide | 1.08 (0.86, 1.35) | 1.15 (0.90, 1.48) | 0.2543 |
| Gliclazide | 0.53 (0.43, 0.64) | 0.86 (0.71, 1.05) | 0.1327 |
| Glimepiride | 0.68 (0.58, 0.79) | 1.08 (0.92, 1.28) | 0.3584 |

**Abbreviations:** HR: hazard ratio, CI: confidence interval, MACEs: major adverse cardiovascular events (a composite of cardiovascular disease events including hospitalizations for ischemic stroke, myocardial infarction, and heart failure), DPP4i: dipeptidyl peptidase-4 inhibitors.

*Adjusted hazard ratios were estimated from the Cox models adjusted for age, sex, diabetes duration, comorbidity history (hypertension, hyperlipidemia, coronary artery diseases, stroke, myocardial infarction, heart failure, Charlson comorbidity index), diabetic complications (via adapted diabetic complication severity index), co-medications ($\alpha-$blockers, $\beta-$blockers, diuretics, calcium channel blockers, angiotensin-II-converting enzyme inhibitors/angiotensin receptor blockers, lipid-lowering agents, anti-platelet agents/anticoagulants, nitroglycerin, digoxin)

**Table S3: Hazards ratios of cardiovascular diseases as compared individual DPP4i with sitagliptin as reference**

| **MACEs**  **(sitagliptin as reference)** | **Unadjusted HR (95% CI)** | **Adjusted HR***  **(95% CI)** | *p* value |
| --- | --- | --- | --- |
| vildagliptin | 0.89 (0.68, 1.18) | 0.97 (0.74, 1.27) | 0.827 |
| saxagliptin | 1.29 (0.99, 1.66) | 1.28 (0.99, 1.65) | 0.062 |
| linagliptin | 1.59 (1.13, 2.22) | 1.28 (0.91, 1.79) | 0.161 |
| **Heart failure**  **(sitagliptin as reference)** | **Unadjusted HR (95% CI)** | **Adjusted HR*  (95% CI)** | *p* value |
| vildagliptin | 0.62 (0.37, 1.03) | 0.73 (0.44, 1.21) | 0.2201 |
| saxagliptin | 1.15 (0.75, 1.76) | 1.14 (0.73, 1.77) | 0.5669 |
| linagliptin | 1.19 (0.68, 2.08) | 0.87 (0.49, 1.56) | 0.6447 |

Abbreviations: HR: hazard ratio, CI: confidence interval, MACEs: major adverse cardiovascular events (a composite of cardiovascular disease events including hospitalizations for ischemic stroke, myocardial infarction, and heart failure), DPP4i: dipeptidyl peptidase-4 inhibitors.

*Adjusted hazard ratios were estimated from the Cox models adjusted for age, sex, diabetes duration, comorbidity history (hypertension, hyperlipidemia, coronary artery diseases, stroke, myocardial infarction, heart failure, Charlson comorbidity index), diabetic complications (via adapted diabetic complication severity index), co-medications ($\alpha-$blockers, $\beta-$blockers, diuretics, calcium channel blockers, angiotensin-II-converting enzyme inhibitors/angiotensin receptor blockers, lipid-lowering agents, anti-platelet agents/anticoagulants, nitroglycerin, digoxin).
